# Analysis of electronic health record data of hepatitis B virus (HBV) patients in primary care: hepatocellular carcinoma (HCC) risk associated with socioeconomic deprivation

**DOI:** 10.1016/j.puhe.2023.10.036

**Published:** 2023-12-12

**Authors:** C. Campbell, T. Wang, I. Gillespie, E. Barnes, P.C. Matthews

**Affiliations:** 1 Nuffield Department of Medicine, University of Oxford, Oxford, UK; 2 NIHR Oxford Biomedical Research Centre, Oxford, UK; 3 GSK, Stevenage, UK; 4 Department of Hepatology, Oxford University Hospitals, John Radcliffe Hospital, Headley Way, Headington, Oxford OX3 9DU, UK; 5 The Francis Crick Institute, 1 Midland Road, London NW1 1AT, UK; 6 Division of Infection and Immunity, University College London, Gower Street, London WC1E 6BT, UK; 7 Department of Infectious Diseases, University College London Hospital, Euston Road, London NW1 2BU, UK

## Abstract

**Objectives:**

We set out to characterise chronic Hepatitis B (CHB) in the primary care population in England and investigate risk factors for progression to hepatocellular carcinoma (HCC).

**Study design:**

Retrospective cohort study.

**Methods:**

We identified 8039 individuals with CHB in individuals aged ≥18 years between 1999-2019 in the English primary care database QResearch. HCC risk factors were investigated using Cox proportional hazards modelling.

**Results:**

Most of those with a record of CHB were males (60%) of non-White ethnicity (>70%), and a high proportion were in the most deprived Townsend deprivation quintile (44%). Among 7029 individuals with longitudinal data, 161 HCC cases occurred. Increased HCC hazards significantly associated with male sex (adjusted hazards ratio (aHR) 3.17, 95% Confidence Interval (95CI) 1.92-5.23), older age (for age groups 56-55 and ≥66 years of age, compared to 26-35 years, aHRs 2.82 (95CI 1.45-5.46) and 3.76 (95CI 1.79-7.9) respectively), Caribbean ethnicity (aHR 3.32, 95CI 1.43-7.71, compared to White ethnicity), ascites (aHR 3.15, 95CI 1.30-7.67), cirrhosis (aHR 6.55, 95CI 4.57-9.38) and peptic ulcer disease (aHR 2.26, 95CI 1.45-3.51).

**Conclusions:**

Targeting interventions and HCC surveillance at vulnerable groups is essential to improve CHB outcomes, and to support progress towards international goals for the elimination of hepatitis infection as a public health threat.

## Introductory Statement

The 2022 Global Burden of Disease analysis of hepatitis B virus (HBV) epidemiology estimates that >300 million people live with chronic infection worldwide (1). Through its progression to cirrhosis and primary liver cancer (hepatocellular carcinoma (HCC)), chronic hepatitis B (CHB) is the leading global cause of HCC death (2), and the third leading cause of death amongst people with cirrhosis (1). Over recent decades, age-standardised death rates have remained constant or increased for HCC and cirrhosis, respectively, and HBV-attributable deaths have increased worldwide (3). International targets calling for the elimination of HBV infection as a public health threat by the year 2030 have been set (4), with recent recognition and investment into the early detection and treatment of HCC (5). Meeting elimination targets requires clear understanding of the epidemiology of infection and associated liver disease in order to target resources and interventions to high-risk groups and benchmark progress.

CHB prevalence has not been robustly estimated in many settings, including the UK (6), and groups at the highest risk of morbidity and mortality have not been well characterised. Furthermore, even in well-defined CHB populations, treatment coverage and eligibility are often unreported. Regional HBV reports from UK public health services (UK Health Security Agency, previously Public Health England) have included neither overall estimates of the proportion of CHB individuals receiving antiviral treatment, nor estimates stratified by relevant subgroups other than age, sex and ethnicity (6–10).

There has been increasing interest in identifying risk factors for CHB progression to cirrhosis, HCC and other endpoints (11). Age, sex, HBV DNA viral load (VL) and viral genotype are established determinants of HCC risk (12–18), and recent studies have reported associations between HCC and various comorbidities, including type 2 diabetes mellitus (T2DM) and hypertension (11). However, few cohorts have been characterised in European countries and/or in ethnically diverse populations, to validate or inform scoring approaches.

Studies based on electronic health records (EHRs) enable characterisation of large retrospective cohorts, thus enhancing statistical power, and identifying a study sample that is more representative of the whole disease population compared to clinical trials. Such databases often have longitudinal follow-up, with exposures and outcomes ascertained over time. EHR databases can often be linked to other registries (such as national cancer registries and vital statistics), allowing for identification of relevant endpoints.

Given the substantial evidence gaps concerning HBV epidemiology, disease burden and risk factors for progression to HCC, we set out to identify a cohort from a large-scale primary care EHR database in England (19) with two aims (i) to characterise the CHB population and (ii) to investigate risk factors for progression to HCC.

## Patients and Methods

### Data source and study population/design

We used data from the England primary care database QResearch (version 45), which contains >35 million patient records from >1800 individual practices (20). QResearch was established in 2002 and contains anonymised individual-level patient EHRs. Data are collected prospectively and are linked to hospital episode statistics (HES), National Cancer Registration Analysis Service (NCRAS) and Office for National Statistics (ONS) mortality data.

We identified individuals from the QResearch database who (at any time between 01 January 1999 and 31 December 2019) were age ≥18 years and had a record of CHB based on either:

(i) diagnostic Systemised Nomenclature of Medicine (SNOMED)/Read or International Classification of Disease (ICD) code indicating CHB (21,22), or
(ii) presence of detectable hepatitis B surface antigen (HBsAg) or HBV DNA (VL) measurement on at least two recordings ≥ 6 months apart (Supplementary Figure 1).

### Covariate selection and ascertainment

We identified relevant covariates for extraction *a priori* (protocol submitted to QResearch) based on previous literature (11,23–30) and clinical relevance. Ethnicity is categorised in QResearch as per 2011 census categories (31). Baseline patient-level Townsend Deprivation quintile is available as a measure of socioeconomic status in QResearch electronic records, and is a multifactorial measure of deprivation which accounts for employment, home and car ownership and domestic overcrowding.

We characterised lifestyle factors, demographics and relevant numeric biomarkers from relevant SNOMED/Read codes. We collected comorbiditiy data from relevant SNOMED/Read and ICD-9 and -10 codes and amalgamated subtypes of comorbid cardiovascular diseases (including ischaemic heart disease, hypertension and cerebrovascular disease) into a single variable to improve model fit. Body mass index (BMI, kg/m^2^) was categorised (underweight, <18.5 kg/m^2^; healthy weight, 18.5-2.49 kg/m^2^; overweight, 25.0-29.9 kg/m^2^; obese, ≥ 30 kg/m^2^) based on World Health Organization (WHO) categories (32). Covariate measurements made within ±3 years of earliest CHB diagnosis and before HCC diagnosis were used as proxy baseline measurements. Where patients had >1 measurement taken within 3 years of the earliest CHB diagnosis, we used measurements taken closest to diagnosis date.

### Outcome ascertainment

Our primary endpoint of interest was HCC, which we ascertained via identification of patients with relevant SNOMED/Read or ICD codes corresponding to HCC, and by linkage of the cohort to National Cancer Registry data (33,34). In order to maximise outcome ascertainment, we used a broad definition for HCC including multiple relevant codes (Supplementary Table 1). We performed sensitivity analysis (further details below) whereby all patients with non-HCC neoplasms were excluded, to investigate robustness of main analysis using our broad HCC definition. A tabulation of HCC cases across source of diagnosis is presented in Supplementary Table 2.

### Follow-up

Earliest date of CHB diagnosis was regarded as cohort entry and initiation of follow-up for each individual. For patients who developed HCC, date of HCC diagnosis was regarded as the end of follow-up. For patients who did not develop HCC (i.e., patients who were censored), follow-up ended at patient cohort exit date (either due to leaving their general practice and switching to a practice which does not contribute to QResearch, or death) or 31 December 2019, whichever occurred earlier. Patients in whom database exit date preceded or was equal to first recorded CHB diagnosis date (n = 1010) whereby follow-up time ≤0 years were excluded from longitudinal analysis.

In some patients, HCC diagnosis date or cohort exit date preceded or was equal to CHB diagnosis date (Supplementary Table 3). Data from these patients were excluded from analyses of HCC risk factors.

### Statistical analysis

Statistical analyses were carried out in R (version 4.1.0). Baseline characteristics were summarised for all CHB patients (regardless of length of follow-up) using descriptive statistics. Means and standard deviations (SDs) or medians and interquartile ranges (IQRs) were presented for continuous measures, and were compared using *t* or Wilcoxon rank-sum tests, respectively. Patient counts and percentages were presented for categorical and binary variables, and were compared using chi-squared or Fisher’s exact tests.

We used univariable and multivariable Cox proportional hazards models to investigate risk factors for progression of CHB to HCC, including variables in the multivariable model based on significance of univariable associations (where *P* ≤0.1) and/or based on biological/clinical relevance and previous literature (11,23–30). A previous meta-analysis we undertook to investigate risk factors for HCC in CHB was also used to inform variable selection (11). Satisfaction of the proportional hazards assumption was assessed by visualisation of log-log Kaplan Meier survival estimates curves. Where the assumption was violated, time-varying covariates were fitted.

Continuous laboratory parameters which were right-skewed were transformed with a natural logarithm for inclusion in multivariable models. Laboratory parameters were divided into quintiles for inclusion in multivariable models. Means and SDs for log AST, log alanine transaminase (ALT) and platelet count (Plt) quintiles are presented in supplement (Supplementary Table 4). Hazard ratios and 95% confidence intervals (95% CI) were reported for Cox proportional hazards model outputs. Analysis on the imputed dataset was used for main models.

### Handling of missing data

Values were missing for Townsend Deprivation Quintile, ethnicity, alcohol consumption, cigarette consumption, BMI, Plt, ALT measurement, aspartate transaminase (AST) measurement, Hepatitis B surface antigen (HBsAg), and HBV viral load (VL). Missing data are described further in Supplementary Table 5.

Multiple imputation by chained equations (MICE) was used to impute missing data across patient characteristics. The assumption of missing at random was made for imputed variables. This is in accordance with previous handling of missing data in cohorts utilising QResearch data (35–38), and current recommendations for imputation of missing data (39). Characteristics with >90% missingness were not imputed. Ten imputed datasets were generated, and results from univariable and multivariable Cox proportional hazards models from each dataset were pooled according to Rubin’s rules (40,41).

### Sensitivity analyses

To test robustness of our main model, we performed three sensitivity analyses (Supplementary Table 6), as follows (i) main results model fit to complete-case cohort subset (i.e. the subset of patients with completeness for all variables); (ii) exclusion of patients with history of non-HCC neoplasms (presented in Supplementary Table 7) to control for unmeasured outcome misclassification whereby secondary liver cancer has been misclassified as primary HCC; (iii) addition of ALT, AST and Plt in the main model fit to the imputed dataset, as the percentage of missingness in these exposures was too high for them to be included in main analysis.

In order to further investigate the association of antiviral therapy with HCC risk, propensity score analysis was undertaken as an additional sensitivity analysis. Specifically a propensity score for initiating antiviral treatment was generated by regressing the odds of treatment initiation onto the following predictors of treatment initiation: age, sex, socioeconomic status, ethnicity, BMI, T2DM, alcohol-related liver disease, cirrhosis, end-stage liver disease and non-alcoholic fatty liver disease. Accordingly two models were fitted to investigate how the association of antiviral therapy with HCC risk changed before and after the addition of the propensity score to the model, in order to determine whether the association of antiviral therapy initiation is confounded by factors associated with treatment initiation.

## Results

### Individuals with CHB are under-represented in primary care records, with the majority of patients with CHB being male, of non-white ethnicity, socioeconomically deprived and untreated

We identified 8039 individuals living with CHB in the QResearch database between 1999 and 2019 from a database-wide denominator of ~35 million individuals, translating to a prevalence of diagnosed CHB of 0.023%. Most of these were identified by a SNOMED/Read or ICD CHB diagnostic code (7856/8039, 97.7%), with a remaining 2.3% (252/8039) identified by HBsAg and/or VL measurements (in the absence of a diagnostic code) (Table 1). Median follow-up duration was 3.87 years (IQR 6.30 years), with differential follow-up between individuals who developed HCC (median follow-up 1.47 years, IQR 5.13 years) and those who did not (median follow-up duration 3.93 years, IQR 6.28 years). Mean age at baseline was 38.3 years (SD 11.6 years), and at baseline >75% were ≤45 years of age. The majority were male (4856/8039, 60.4%).

**Figure 1:**
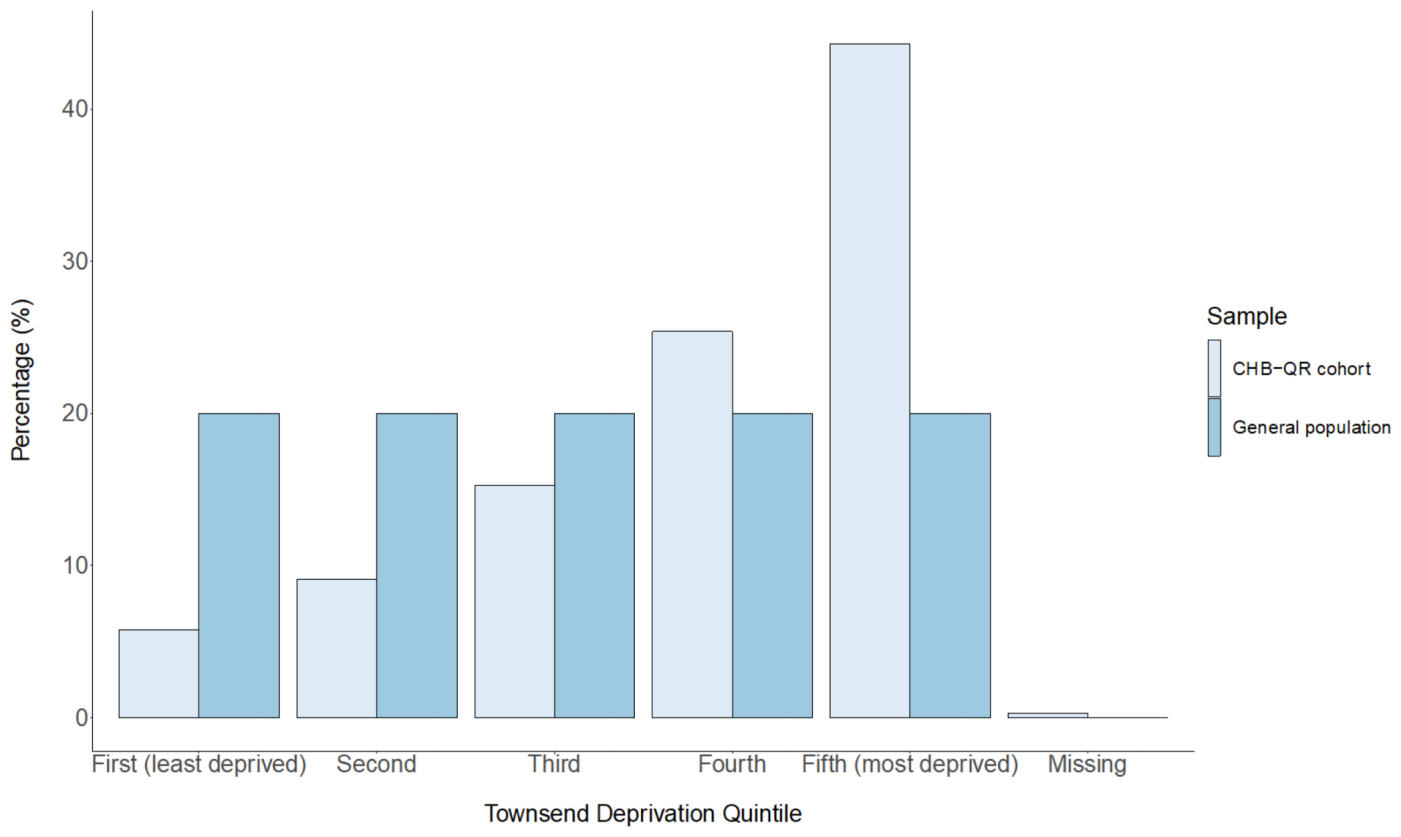
Townsend deprivation quintile breakdown in 8039 adults with chronic hepatitis B virus infection derived from the QResearch primary care database (England) versus the United Kingdom general population. CHB-QR, individuals with chronic hepatitis b in the QResearch primary care database.

**Figure 2:**
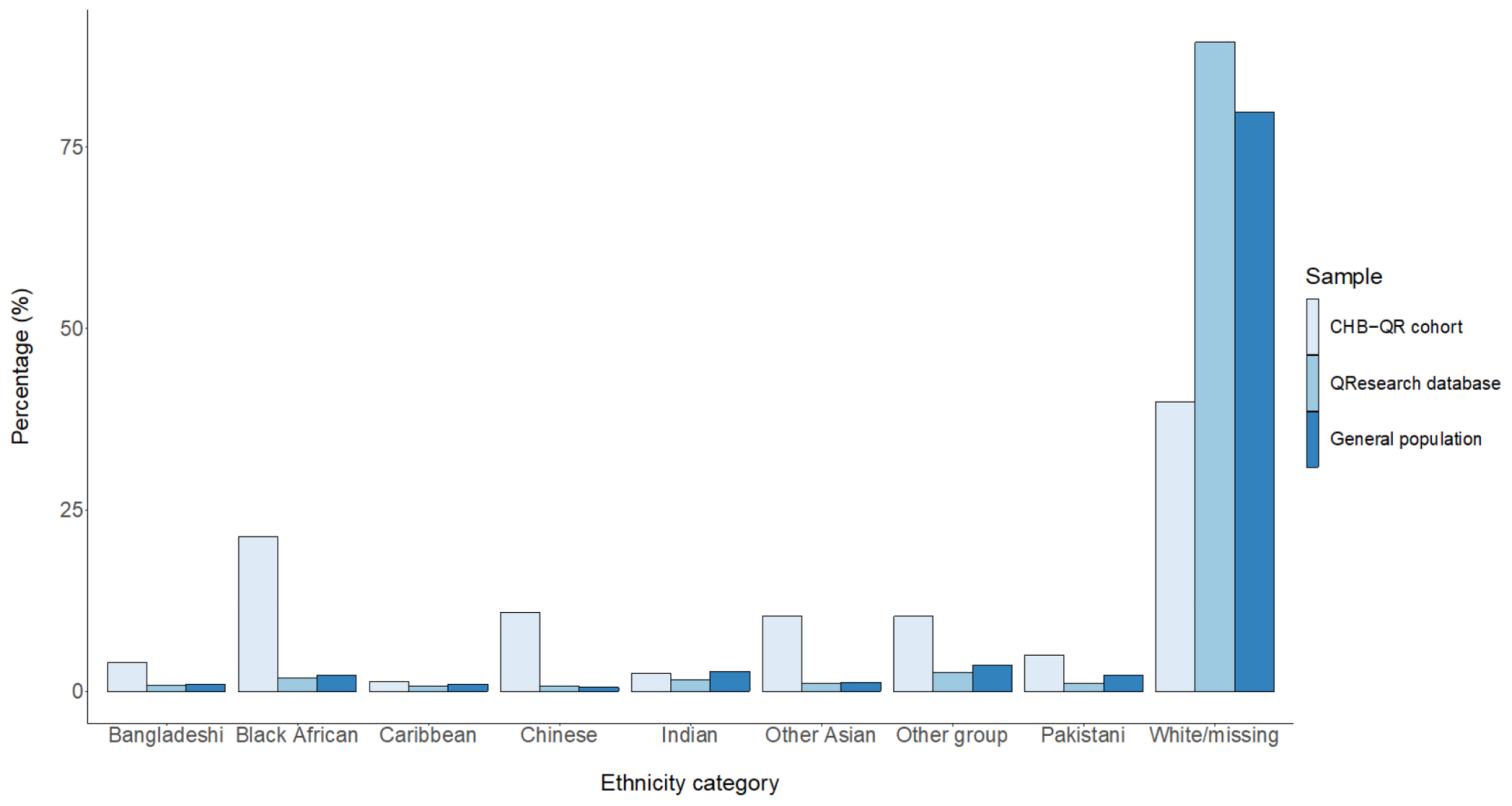
Ethnicity breakdown in 8039 adults in a chronic hepatitis B virus cohort characterised from the QResearch primary care database (England) versus all individuals in the QResearch database (~35 million), vs. the United Kingdom general population. General population estimates obtained from 2019 estimates from the Office for National Statistics. CHB-QR, individuals with chronic hepatitis b in the QResearch primary care database.

Black African ethnicity represented 25.4% of individuals, with 12.9% of Chinese ethnicity, 5.9% of Pakistani ethnicity, 3.0% of Indian ethnicity and 28.4% of White ethnicity (Table 1). Proportions of Black and ethnic minorities in our CHB cohort were greater than those in both the wider QResearch database (42) and general English population (43) (Figures 1,2). Data for smoking consumption, alcohol consumption and BMI were available for 71.5%, 56.1% and 61.3% of the cohort (Table 1). At baseline, most individuals (88.0%) had no record of antiviral treatment. Within 1, 1-2, 2-3 and ≥ 4 years of CHB diagnoses, cumulatively 2.7%, 4.0 %, 5.0% and 10.1% of patients had record of antiviral treatment initiation from baseline, respectively.

Age and sex were significantly associated with Townsend deprivation quintile, with more deprived quintiles characterised by younger mean age (*P* < 0.001) and higher proportions of males (*P* = 0.021) (Table 2). Ethnicity differed across quintiles (*P* < 0.001) whereby the proportions of Bangladeshi and Black African ethnicity patients increased with increasing deprivation, and proportions of White and Chinese patients decreased. Alcohol and cigarette consumption were also associated with deprivation quintile (*P* < 0.001 and *P* = 0.04, respectively), but no obvious trends were apparent across quintiles. No associations of antiviral treatment, antidiabetic drug, antihypertensive, NSAID and statin use with deprivation quintile were observed.

### Prevalence of diabetes and hypertension in adults with CHB was higher than in the general population

Baseline prevalence of T2DM and hypertension were 8.9% and 15.3%, respectively, differing from prevalences of <8% and <3%, respectively, that have been reported in the wider QResearch cohort representing the UK population (44). Prevalence of other comorbidities (including congestive heart failure, chronic kidney disease, alcohol-related liver disease, ascites, autoimmune hepatitis, cerebrovascular disease, end-stage liver disease, ischaemic heart disease, no-alcoholic fatty liver disease and peptic ulcer disease; Table 1) ranged from 0.1% to 5%. A minority (8.6%) of the cohort had a diagnostic code indicating cirrhosis. Frequency of medication use was as follows: antidiabetic drugs (9.2%), antihypertensives (5.1%), non-steroidal anti-inflammatory drugs (NSAIDs) (5.9%) and statins (5.7%). Prevalence of non-HCC neoplasm was 4.7% in the overall cohort.

### Risk factors for HCC included male sex, older age, increased deprivation, Caribbean ethnicity and peptic ulcer disease

**Figure 3:**
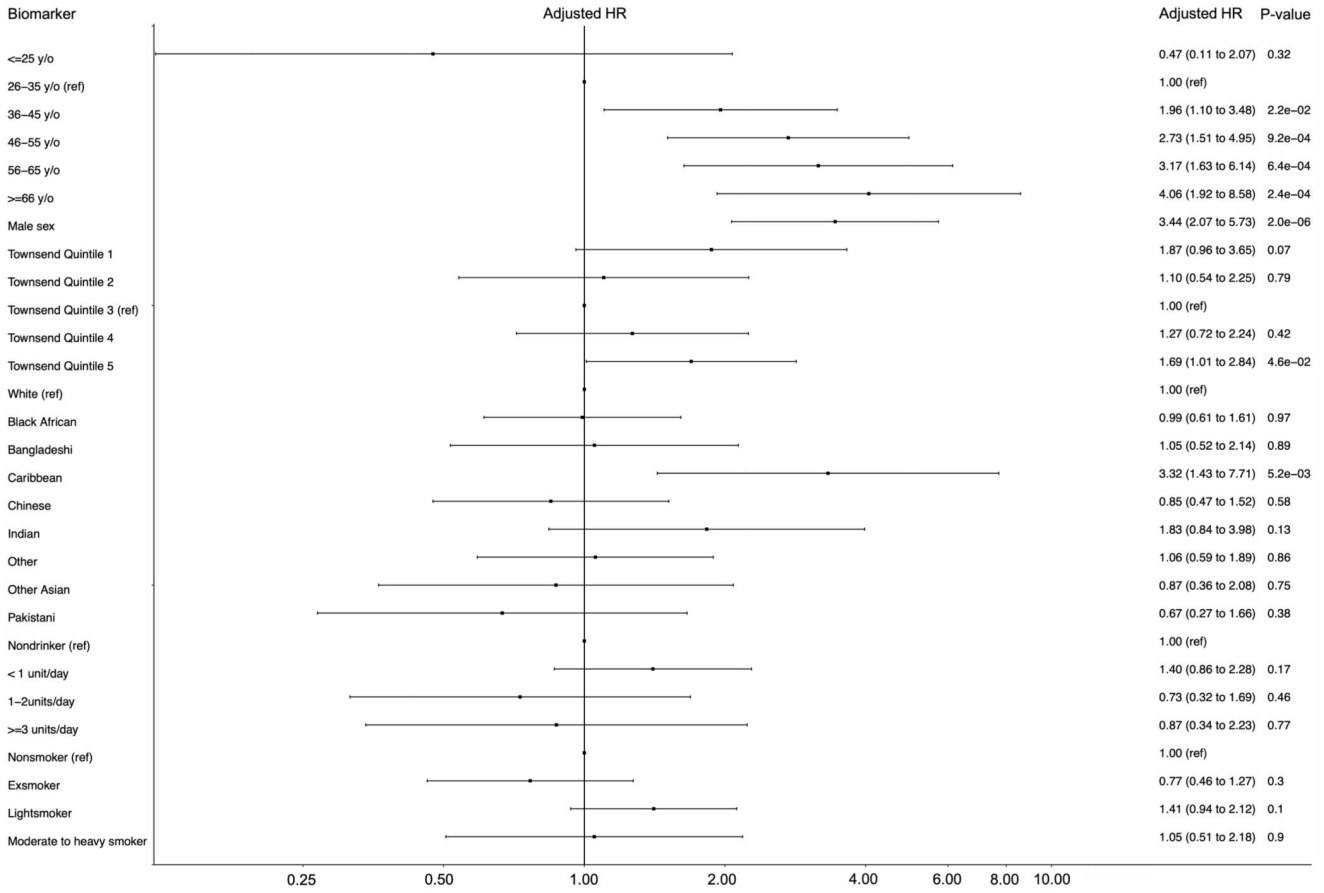

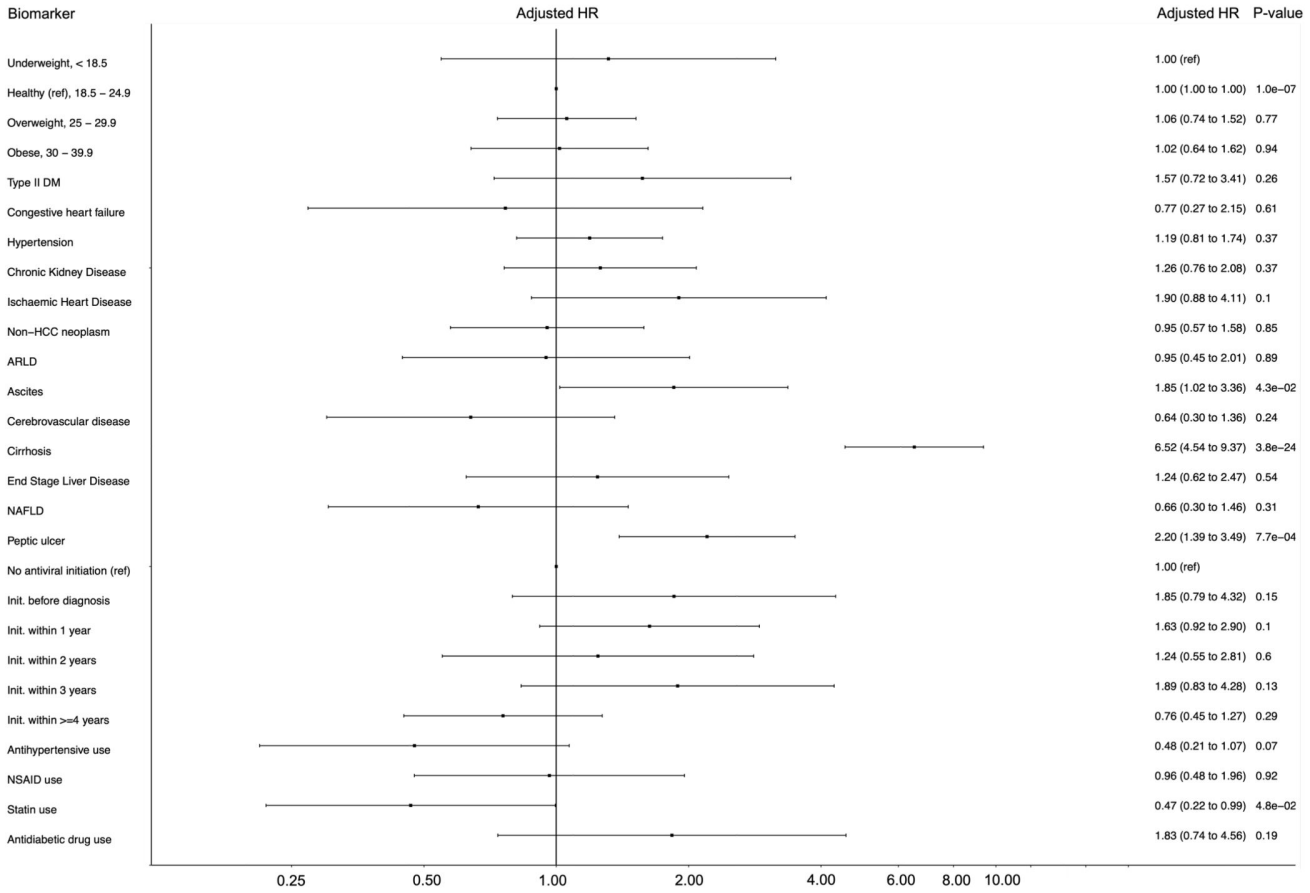
Forest plot for Cox proportional hazards model to identify risks of hepatocellular carcinoma (HCC) in an adult population with chronic Hepatitis B virus infection derived from the QResearch primary care database. Analysed using a dataset generated by multiple imputation with chained equations (n = 7029, HCC cases = 161). First (least deprived) to fifth (most deprived) Townsend Deprivation Quintiles are denoted by SES1-5, respectively. ‘Init.’ refers to treatment initiation with antiviral therapy.

Baseline characteristics of the imputed dataset used in analysis of HCC risk factors are presented in Supplementary Table 8. Multivariable Cox proportional hazards models were constructed for 7029 patients in whom 161 HCC cases developed throughout 41,147 person-years of follow-up (Figure 3, Table 3). This translated to an HCC incidence rate of 5.10 cases per 1000 person-years (95% Confidence Interval (95CI) 4.46 to 5.84).

Hazards of HCC were increased in males (adjusted hazards ratio (aHR) 3.17, 95% CI 1.92 to 5.23), with increasing age (aHR for 36-45 years 1.92, 95% CI 1.08 to 3.42; aHR for 46-55 years 2.63, 95% CI 1.46 to 4.76; aHR for 56-65 years 2.82, 95% CI 1.45 to 5.46; aHR for ≥66 years 3.76, 95% CI 1.79 to 7.90, as compared to 26-35 years reference group) and in the fifth deprivation quintile as compared to the third quintile (aHR 1.69, 95% CI 1.01 to 2.84). Hazards of HCC in the Caribbean ethnicity group were higher than those in the White reference group (aHR 3.15, 95% CI 1.30 to 7.67) but did not differ in any other ethnic category. There were no associations between alcohol consumption, cigarette smoking, or BMI with hazards of HCC. As expected, increased hazards of HCC were associated with evidence of advanced liver disease cirrhosis (aHR 6.55, 95% CI 4.57 to 9.38) and ascites (aHR 2.28, 95% CI 1.28 to 4.06). Interestingly, peptic ulcer disease was also associated with increased HCC hazards (aHR 2.26, 95% CI 1.45 to 3.51). No medicines, including antiviral treatment, associated with hazards of HCC. however it is important to note that statin use was associated with reduced hazards of HCC, although confidence intervals for this association crossed the null. Kaplan Meier curves for the associations of ascites, cirrhosis and peptic ulcer disease are available in Supplementary Figure 2.

Hazards ratios did not change materially in strength or direction upon sensitivity analysis excluding non-HCC neoplasms or including AST, ALT and Plt at baseline (Table 3).

### Interrogation of association of antiviral therapy with hazards of HCC

To interrogate the association of antiviral therapy with increased hazards of HCC in our main model results (Table 3), we undertook sensitivity analysis whereby a propensity score for initiating antiviral treatment was generated. We fitted two models to investigate how the association of antiviral therapy with HCC risk changed before and after the addition of the propensity score (Supplementary Table 9). Before addition of the propensity score to the model, antiviral therapy associated with an increased HCC risk, likely reflecting shared factors associating with both antiviral treatment initiation and increased HCC risk such as increasing age, male sex and comorbid liver disease. Following addition of the propensity score to the model, this association of antiviral therapy with increased HCC risk was attenuated towards the null and 95% CI crossed 1.00.

### Main model results are robust to complete-case sensitivity analysis

We undertook sensitivity analysis restricted to the subgroup of patients for whom complete data were available (n=3648 patients in whom 68 cases of HCC occurred (Supplementary Table 10)). Hazard ratios did not change materially in strength or direction in sensitivity analysis undertaken to exclude patients with history of non-HCC neoplasms (Supplementary Table 10).

## Discussion

### Summary of key findings

This is the largest population of individuals living with HBV characterised in England to date, from either EHR or traditional prospective cohorts. Our CHB group was ethnically diverse, with higher proportions of black and ethnic minority individuals than the total QResearch database (42) or general English population (43). The CHB cohort is disproportionately socioeconomically deprived, with substantial burdens of comorbid disease. We identified increased hazards of HCC associated with increasing age, male sex, socioeconomic deprivation, Caribbean ethnicity, severe liver disease (ascites and cirrhosis), and comorbid disease (peptic ulcer disease). We report a protective association of statin use with HCC risk. Although antiviral treatment is known to moderate HCC risk (45–47), this association was not identified in this dataset. Age, sex and T2DM have previously been found to positively associate with HCC risk in CHB (11), however this is the first study to confirm these associations in an ethnically diverse cohort. The QResearch database has geographic coverage across England, therefore findings should be generalizable across the country, but thorough representation is precluded as many individuals living with CHB are either undiagnosed or not represented in primary care EHR.

### Influence of HBV genotype

HBV genotype is not routinely determined in clinical practice and therefore not available in EHRs. Viral genotypes associate with ethnicity (48,49), increased HCC risks (50) and antiviral treatment resistance (51). Therefore, associations of HCC risk with ethnicity (and thereby socioeconomic deprivation) may be confounded by genotype or mediated by unmeasured population genetic or lifestyle factors.

### Drug treatment and HCC development

A protective association of statin use with HCC hazards has been reported in previous CHB cohorts (11,52,53), in individuals with predisposing HCC risk factors including cirrhosis and T2DM (54,55) and a general patient population (53–55). However, this association may also be confounded by health-seeking behaviour and/or healthcare engagement whereby unmeasured lifestyle factors or healthcare interventions associate with both statin use and reduced HCC risk. Further analysis, including mediation analysis where data allows, is warranted to investigate potential mechanisms. Similarly, the positive association of peptic ulcer disease with hazards of HCC may be confounded by proton pump inhibitor (PPI) administration for peptic ulcer treatment. PPI prescription/usage was not available in our sample, but previous observational studies report increased risks of HCC with PPIs (56,57). Pooled risk estimates from meta-analyses are variable (58–60). It is possible that this association can be attributed to ascertainment bias and is confounded by cirrhosis, whereby cirrhotic patients are more likely to undergo surveillance endoscopy and thereby have peptic ulcer disease detected more frequently than non-cirrhotic patients.

Unlike previous observational and randomised interventional studies providing evidence that treatment with nucleoside analogues (NAs) reduces HCC risk (45–47), we do not report this association. Throughout our study, only 10.3% of individuals were documented to have initiated treatment. However, treatment data may be missing from primary care records, as HBV prescribing is based in secondary/tertiary care (61), or a low proportion of our primary care population may be treatment eligible. It is also feasible that our follow-up periods are too short for us to be able to identify a signal for the protective effects of antiviral therapy, which is a long-term intervention.

### Application of data to HCC risk stratification

HCC risk scores (including PAGE-B (24), REACH-B (25,62), GAG-HCC (26,27) and CU-HCC (28–30)) incorporate various characteristics, including age, sex, and laboratory parameters to predict HCC risk. The utility of existing risk scores in homogenous patient subgroups has been demonstrated (63). Future analyses are required to validate (and potentially modify) scores in heterogenous ethnically and clinically diverse samples to inform interventions.

### Limitations of primary care EHR analysis – missing data

We report substantial data missingness, due to poor primary care access/coverage HBV. The crude prevalence estimate for HBV in this primary care dataset (0.023%) underestimates population prevalence by at least an order of magnitude, as recent estimates for prevalence of HBV in the UK suggest a prevalence of approximately 0.5% (64). Missingness is likely associated with unmeasured characteristics, therefore marginalised subgroups (including undocumented migrants, highly mobile population subgroups, and people who do not speak English) may be under-represented.

Most patients were identified by coding, with a minority (3.0%) having confirmatory laboratory tests accessible in QResearch. This is logical as specialist referral is recommended following HBsAg positivity (65) and second confirmatory tests are performed in secondary/tertiary care. Poor linkage between primary and secondary care health data is currently a missed opportunity for high quality clinical service provision, and for translational health research. Enhanced data linkage between primary and secondary care would provide direct benefits for overall patient management with diverse clinical and public health benefits. For HBV infection specifically, such linkage would improve the quality of national data, assist with screening and prevention interventions, enhance linkage to services and continuity of care, and provide opportunities for improved early diagnosis of liver complications (including HCC).

Our median follow-up is relatively short for a chronic disease, despite a 20-year study period. However time lags between notification of patient characteristic/disease and input into electronic systems is common in EHR databases. Additionally it is likely that individuals living with HBV infection present to primary care late in infection course and/or notification of secondary/tertiary care infection management is not linked to primary care EHR. Differential follow-up between individuals with and without HCC may be due to late HCC presentation with advanced symptomatic liver disease.

Improved characterisation of relevant variables which may influence HCC risk in primary care EHR would improve quality of future analyses. For example, robust identification of cirrhosis would enable stratification according to this disease subgroup, and would allow for interrogation of underlying disease mechanisms for other risk factors. At present, cirrhosis is poorly coded due to heterogeneity of disease phenptype, diverse underlying aetiology, varied approaches to investigation, and lack of robust and universal case definitions. In addition, further research into potential nutritional HCC risk factors, including the effect of underweight status, obesity and alcohol consumption, would provide additional insights into preventive strategies and patient management. This is especially relevant to the HBV population as nutritional habits may associate with socioeconomic characteristics.

### Analytical corrections for missing data

Imputation of missing baseline data was undertaken in line with previous QResearch investigations (35–38). We were unable to impute HBV biomarkers (including VL and HBsAg) as >90% of participants missed baseline measurement. We therefore excluded these variables from analysis. High missingness was observed for relevant biomarkers (AST, ALT and Plt) indicative of liver health, which are used to score fibrosis and cirrhosis stage and we therefore could not validate/modify HCC risk scores. Analysis in more complete secondary care datasets is warranted to determine the utility of laboratory parameters as robust predictors of disease endpoints and estimate effect sizes. Missingness of biopsy, imaging, or laboratory scores in primary care EHR (66) limited cirrhosis identification, thereby underestimating the prevalence in the cohort and preventing robust investigation of cirrhosis as an endpoint. Similarly prevalence of other comorbidities are likely underestimated, and we are underpowered to detect associations with HCC risk.

Many participants are missing alcohol and cigarette consumption data, and complete measurements may systematically underestimate intake based on self-reporting bias. Although consumption may associate with HCC risk, we cannot report this association in our study. We were also unable to time-update models for changes in alcohol and cigarette consumption throughout follow-up due to lack of repeated measurements (>90% of individuals had one-off records).

### Impact and recommendations

Our results demonstrate that the burden of CHB in the UK is concentrated in a young, ethnically diverse and socioeconomically deprived disease population. Improving access to clinical services, routine HCC surveillance and screening coverage, and representation in large-scale national datasets is necessary. This is warranted not only to improve patient outcomes and reduce the attributable disease burden, but also to obtain more representative data from which more mechanistic and causal inference insights may be gleaned in order to identify specific opportunities for intervention along the patient pathway. Improved linkage of primary and secondary care EHR datasets is essential to achieve these goals.

### Conclusions

The CHB population in England is ethnically diverse and socioeconomically deprived. We identified risk factors for HCC, and validated associations observed in previous CHB cohorts. Missingness limits identification of CHB individuals and robust description of those identified. Improved data capture by EHR systems, and enhanced communication between primary and secondary care records, is crucial to provide an evidence base for interventions, including diagnostic screening, treatment and surveillance, modification of risk factors for HCC, and monitoring progress towards elimination targets.

## Supplementary Material

Supplementary Figure 1

## Figures and Tables

**Table 1:** Cohort baseline characteristics

Characteristic	Total, n (%)	HCC cases, n (%)	Non-HCC cases, n (%)
N	8039	210	7829
Follow-up time (years), median (IQR)	3.87 (6.30)	1.47 (5.13)	3.93 (6.28)
CHB diagnosis source, n (%) *			
Any ICD	3952 (49.2)	47 (22.4)	3789 (48.4)
Any SNOMED/Read	4425 (55.0)	68 (32.4)	4357 (55.7)
Any laboratory test	240 (3.0)	<5	238 (3.0)
Age (years), mean (SD)	38.3 (11.6)	50.3 (12.4)	37.9 (11.4)
Age group, n (%)			
18-25 years	860 (10.7)	<5	857 (10.9)
26-35 years	2930 (36.4)	22 (10.5)	2908 (37.1)
36-45 years	2346 (29.2)	52 (24.8)	2294 (29.3)
46-55 years	1182 (14.7)	63 (30.0)	1119 (14.3)
56-65 years	502 (6.2)	41 (19.5)	461 (5.9)
≥66 years	219 (2.7)	29 (13.8)	190 (2.4)
Sex, n (%)			
Female	3183 (39.6)	24 (11.4)	3159 (40.3)
Male	4856 (60.4)	186 (88.6)	4670 (59.7)
Townsend Deprivation Quintile, n (%)			
First (least deprived)	467 (5.8)	27 (12.9)	440 (5.6)
Second	731 (9.1)	20 (9.5)	711 (9.1)
Third	1226 (15.3)	27 (12.9)	1199 (15.3)
Fourth	2040 (25.4)	48 (22.9)	1992 (25.4)
Fifth (most deprived)	3553 (44.3)	87 (41.4)	3466 (44.3)
Missing	22 (0.3)	<5	21 (0.3)
Ethnicity, n (%)			
Black African	1714 (25.4)	35 (22.3)	1679 (25.5)
Bangladeshi	325 (4.8)	11 (7.0)	314 (4.8)
Caribbean	104 (1.5)	7 (4.5)	97 (1.5)
Chinese	871 (12.9)	21 (13.4)	850 (12.9)
Indian	203 (3.0)	7 (4.5)	196 (3.0)
Other	832 (12.3)	15 (9.6)	817 (12.4)
Other Asian	384 (5.7)	5 (3.2)	379 (5.7)
Pakistani	401 (5.9)	7 (4.5)	394 (6.0)
White	1916 (28.4)	49 (31.2)	1867 (28.3)
Missing	1289 (16.0)	53 (25.2)	1236 (15.8)
Cigarette consumption, n (%)			
Non-smoker	3810 (66.2)	78 (54.2)	3732 (66.5)
Ex-smoker	768 (13.4)	25 (17.4)	743 (13.2)
Light smoker (1-9 cigarettes/day)	908 (15.8)	31 (21.5)	877 (15.6)
Moderate to heavy smoker (≥10 cigarettes per day)	266 (4.6)	10 (6.9)	256 (4.6)
Missing	2287 (28.5)	66 (31.4)	2221 (28.4)
Alcohol consumption, n (%)			
Non-drinker	3697 (81.9)	100 (83.3)	3597 (81.9)
Trivial drinker (<1 unit per day)	485 (10.7)	13 (10.8)	472 (10.7)
Light drinker (1-2 units per day)	209 (4.6)	<5	206 (4.7)
Moderate to heavy drinker (≥3 units per day)	121 (2.7)	<5	117 (2.7)
Missing	3527 (43.9)	90 (42.9)	3439 (43.9)
BMI (kg/m^2^), mean (SD)	26.05 (5.06)	26.13 (4.63)	26.05 (5.07)
BMI, n (%)			
Underweight (<18.5 kg/m^2^)	159 (3.2)	<5	156 (3.2)
Normal weight (18.5-24.9 kg/m^2^)	2092 (42.5)	49 (39.8)	2043 (42.5)
Overweight (25.0-29.9 kg/m^2^)	1734 (35.2)	52 (42.3)	1682 (35.0)
Obese (≥30 kg/m^2^)	943 (19.1)	19 (15.4)	924 (19.2)
Missing	3111 (38.7)	87 (41.4)	3024 (38.6)
Antiviral initiation, n (%)			
No initiation	7076 (88.0)	140 (66.7)	6936 (88.6)
Before CHB diagnosis	138 (1.7)	11 (5.2)	127 (1.6)
Within 1 year of CHB diagnosis	221 (2.7)	24 (11.4)	197 (2.5)
Within 1-2 years of CHB diagnosis	120 (1.5)	7 (3.3)	113 (1.4)
Within 2-3 years of CHB diagnosis	78 (1.0)	7 (3.3)	71 (0.9)
≥4 years after CHB diagnosis	406 (5.1)	21 (10.0)	385 (4.9)
Type 2 DM, n (%)	715 (8.9)	21 (10.0)	664 (8.5)
Congestive heart failure, n (%)	75 (0.9)	7 (3.3)	68 (0.9)
Hypertension, n (%)	1229 (15.3)	71 (33.8)	1158 (14.8)
Chronic kidney disease, n (%)	287 (3.6)	29 (13.8)	258 (3.3)
Alcohol-related liver disease, n (%)	93 (1.2)	19 (9.0)	74 (0.9)
Ascites, n (%)	114 (1.4)	32 (15.2)	82 (1.0)
Autoimmune hepatitis, n (%)	9 (0.1)	<5	9 (0.1)
Cerebrovascular disease, n (%)	310 (3.9)	21 (10.0)	289 (3.7)
Cirrhosis, n (%)	689 (8.6)	121 (57.6)	568 (7.3)
End-stage liver disease, n (%)	85 (1.1)	16 (7.6)	69 (0.9)
Ischaemic heart disease, n (%)	255 (3.2)	20 (9.5)	235 (3.0)
Non-alcoholic fatty liver disease, n (%)	404 (5.0)	8 (3.8)	396 (5.1)
Non-HCC neoplasm, n (%)	381 (4.7 )	36 (17.1)	345 (4.4)
Peptic ulcer, n (%)	248 (3.1)	32 (15.2)	216 (2.8)
Any cardiovascular disease^†^, n (%)	1414 (17.6)	87 (41.4)	1327 (16.9)
Antidiabetic drug use, n (%)	737 (9.2)	49 (23.3)	688 (8.8)
Antihypertensive use, n (%)	408 (5.1)	22 (10.5)	386 (4.9)
NSAID use, n (%)	472 (5.9)	24 (11.4)	448 (5.7)
Statin use, n (%)	457 (5.7)	19 (9.0)	438 (5.6)

CHB, chronic hepatitis B virus infection; ICD, International classification of disease; SNOMED, Systemised Nomenclature of Medicine; SD, standard deviation; BMI, body mass index; DM, diabetes mellitus; HCC, hepatocellular carcinoma; NSAID, non-steroidal anti-inflammatory drug.

* Some patients have diagnostic indicators of CHB from multiple sources and therefore there overlap between categories.

† Includes congestive heart failure, hypertension, cerebrovascular disease and ischaemic heart disease.

**Table 2:** Characteristics of adults with chronic hepatitis B virus infection identified from QResearch primary care database, stratified by socioeconomic status.

	Townsend Deprivation Quintile						
Characteristic	First (least deprived)	Second	Third	Fourth	Fifth (most deprived)	Missing	*P*
N	467	731	1226	2040	3553	22	
Follow-up time (years), mean (SD)	6.33 (6.59)	6.15 (7.65)	5.40 (5.48)	4.94 (5.25)	5.10 (5.20)	3.79 (3.56)	<0.001
HCC cases, n (%)	27 (5.8%)	20 (2.7%)	27 (2.2%)	48 (2.4%)	87 (2.4%)	<5	0.001
Age (years), mean (SD)	43.64 (13.10)	40.11 (12.26)	38.98 (11.91)	37.35 (11.22)	37.45 (11.08)	38.18 (9.52)	<0.001
Age group, n (%)							<0.001
18-25 years	110 (23.6%)	247 (33.8%)	433 (35.3%)	799 (39.2%)	1330 (37.4%)	11 (50.0%)	
26-35 years	25 (5.4%)	64 (8.8%)	112 (9.1%)	244 (12.0%)	415 (11.7%)	<5	
36-45 years	153 (32.8%)	207 (28.3%)	379 (30.9%)	561 (27.5%)	1041 (29.3%)	5 (22.7%)	
46-55 years	86 (18.4%)	124 (17.0%)	178 (14.5%)	281 (13.8%)	509 (14.3%)	<5	
56-65 years	58 (12.4%)	64 (8.8%)	76 (6.2%)	116 (5.7%)	186 (5.2%)	<5	
≥66 years	35 (7.5%)	25 (3.4%)	48 (3.9%)	39 (1.9%)	72 (2.0%)	<5	
Sex, n (%)							0.021
Female	214 (45.8%)	300 (41.0%)	505 (41.2%)	802 (39.3%)	1355 (38.1%)	7 (31.8%)	
Male	253 (54.2%)	431 (59.0%)	721 (58.8%)	1238 (60.7%)	2198 (61.9%)	15 (68.2%)	
Ethnicity, n (%)							<0.001
Black African	26 (7.2%)	69 (11.9%)	155 (15.8%)	386 (22.5%)	1073 (34.6%)	≤5	
Bangladeshi	<5	10 (1.7%)	26 (2.6%)	56 (3.3%)	229 (7.4%)	<5	
Caribbean	<5	<5	14 (1.4%)	35 (2.0%)	49 (1.6%)	<5	
Chinese	84 (23.3%)	121 (20.9%)	156 (15.9%)	201 (11.7%)	305 (9.8%)	<5	
Indian	10 (2.8%)	17 (2.9%)	55 (5.6%)	80 (4.7%)	40 (1.3%)	<5	
Other	49 (13.6%)	58 (10.0%)	93 (9.5%)	216 (12.6%)	415 (13.4%)	<5	
Other Asian	24 (6.7%)	46 (7.9%)	67 (6.8%)	99 (5.8%)	147 (4.7%)	<5	
Pakistani	19 (5.3%)	41 (7.1%)	98 (10.0%)	153 (8.9%)	90 (2.9%)	<5	
White	143 (39.7%)	213 (36.8%)	318 (32.4%)	486 (28.4%)	755 (24.3%)	<5	
Cigarette consumption, n (%)							0.04
Non-smoker	211 (67.8%)	326 (66.3%)	581 (66.3%)	978 (66.4%)	1704 (65.9%)	10 (66.7%)	
Ex-smoker	51 (16.4%)	84 (17.1%)	122 (13.9%)	167 (11.3%)	343 (13.3%)	<5	
Light smoker (1-9 cigarettes/day)	37 (11.9%)	62 (12.6%)	132 (15.1%)	248 (16.8%)	425 (16.4%)	<5	
Moderate to heavy smoker (≥10 cigarettes per day)	12 (3.9%)	20 (4.1%)	41 (4.7%)	80 (5.4%)	113 (4.4%)	<5	
Alcohol consumption, n (%)							<0.001
Non-drinker	183 (81.0%)	263 (76.9%)	553 (82.2%)	959 (82.9%)	1732 (82.3%)	7 (70.0%)	
Trivial drinker (<1 unit per day)	32 (14.2%)	42 (12.3%)	70 (10.4%)	120 (10.4%)	221 (10.5%)	<5	
Light drinker (1-2 units per day)	<5	21 (6.1%)	31 (4.6%)	39 (3.4%)	113 (5.4%)	<5	
Moderate to heavy drinker (≥3 units per day)	8 (3.5%)	16 (4.7%)	19 (2.8%)	39 (3.4%)	38 (1.8%)	<5	
BMI (kg/m^2^), mean (SD)	25.34 (4.76)	25.72 (4.92)	25.81 (4.94)	26.30 (5.31)	26.14 (5.01)	26.56 (4.47)	0.031
BMI, n (%)							0.433
Underweight (<18.5 kg/m^2^)	11 (4.3%)	13 (3.2%)	27 (3.7%)	38 (3.0%)	70 (3.1%)	<5	
Normal weight (18.5-24.9 kg/m^2^)	125 (48.6%)	176 (43.2%)	322 (43.6%)	533 (42.3%)	933 (41.4%)	<5	
Overweight (25.0-29.9 kg/m^2^)	79 (30.7%)	146 (35.9%)	260 (35.2%)	422 (33.5%)	822 (36.5%)	5 (50.0%)	
Obese (≥30 kg/m^2^)	42 (16.3%)	72 (17.7%)	129 (17.5%)	268 (21.3%)	430 (19.1%)	<5	
Antiviral initiation, n							0.445
No initiation	393 (84.2%)	647 (88.5%)	1071 (87.4%)	1808 (88.6%)	3138 (88.3%)	19 (86.4%)	
Before CHB diagnosis	13 (2.8%)	17 (2.3%)	24 (2.0%)	32 (1.6%)	52 (1.5%)	<5	
Within 1 year of CHB diagnosis	19 (4.1%)	16 (2.2%)	39 (3.2%)	46 (2.3%)	100 (2.8%)	<5	
Within 1-2 years of CHB diagnosis	7 (1.5%)	9 (1.2%)	14 (1.1%)	29 (1.4%)	61 (1.7%)	<5	
Within 2-3 years of CHB diagnosis	7 (1.5%)	6 (0.8%)	15 (1.2%)	21 (1.0%)	28 (0.8%)	<5	
≥4 years after CHB diagnosis	28 (6.0%)	36 (4.9%)	63 (5.1%)	104 (5.1%)	17,4 (4.9%)	<5	
Type 2 DM, n (%)	39 (8.4%)	51 (7.0%)	119 (9.7%)	180 (8.8%)	323 (9.1%)	<5	0.383
Congestive heart failure, n (%)	6 (1.3%)	10 (1.4%)	12 (1.0%)	19 (0.9%)	28 (0.8%)	<5	0.675
Hypertension, n (%)	85 (18.2%)	97 (13.3%)	183 (14.9%)	298 (14.6%)	563 (15.8%)	<5	0.212
Chronic kidney disease, n (%)	20 (4.3%)	21 (2.9%)	46 (3.8%)	75 (3.7%)	123 (3.5%)	<5	0.553
Alcohol-related liver disease, n (%)	10 (2.1%)	7 (1.0%)	13 (1.1%)	27 (1.3%)	36 (1.0%)	<5	0.336
Ascites, n (%)	12 (2.6%)	10 (1.4%)	20 (1.6%)	24 (1.2%)	47 (1.3%)	<5	0.188
Autoimmune hepatitis, n (%)	<5	<5	<5	<5	<5	<5	NA
Cerebrovascular disease, n (%)	30 (6.4%)	29 (4.0%)	54 (4.4%)	74 (3.6%)	123 (3.5%)	<5	0.035
End-stage liver disease, n (%)	8 (1.7%)	6 (0.8%)	12 (1.0%)	22 (1.1%)	36 (1.0%)	<5	0.414
Ischaemic heart disease, n (%)	20 (4.3%)	29 (4.0%)	41 (3.3%)	67 (3.3%)	98 (2.8%)	<5	0.28
Non-alcoholic fatty liver disease, n (%)	22 (4.7%)	33 (4.5%)	65 (5.3%)	96 (4.7%)	188 (5.3%)	<5	0.728
Non-HCC neoplasm, n (%)	132 (28.3%)	161 (22.0%)	272 (22.2%)	359 (17.6%)	699 (19.7%)	<5	<0.001
Peptic ulcer, n (%)	26 (5.6%)	13 (1.8%)	43 (3.5%)	58 (2.8%)	108 (3.0%)	<5	0.008
Any cardiovascular disease^†^, n (%)	101 (21.6)	123 (16.8)	218 (17.8)	337 (16.5)	632 (17.8)	<5	0.185
Antidiabetic drug use, n (%)	41 (8.8%)	54 (7.4%)	125 (10.2%)	183 (9.0%)	330 (9.3%)	<5	0.242
Antihypertensive use, n (%)	25 (5.4%)	36 (4.9%)	66 (5.4%)	107 (5.2%)	174 (4.9%)	<5	0.865
NSAID use, n (%)	26 (5.6%)	29 (4.0%)	86 (7.0%)	112 (5.5%)	217 (6.1%)	<5	0.106
Statin use, n (%)	21 (4.5%)	30 (4.1%)	78 (6.4%)	114 (5.6%)	213 (6.0%)	<5	0.268

CHB, chronic hepatitis B virus infection; ICD, International classification of disease; SNOMED, Systemised Nomenclature of Medicine; SD, standard deviation; BMI, body mass index; DM, diabetes mellitus; HCC, hepatocellular carcinoma; NSAID, non-steroidal anti-inflammatory drug.

† Includes congestive heart failutre, hypertension, cerebrovascular disease and ischaemic heart disease.

**Table 3:** Cox proportional hazards model using complete imputed dataset generated by multiple imputation with chained equations (n = 7029, HCC cases = 161).

Characteristic	Univariable HR (95% CI)	Multivariable HR (95% CI)	Multivariable HR sensitivity analysis I (95% CI) †	Multivariable HR sensitivity analysis II (95% CI) ‡
Age group				
18-25 years	0.47 (0.11 to 2.05)	0.45 (0.1 to 1.98)	0.41 (0.09 to 1.82)	0.58 (0.13 to 2.7)
26-35 years	1.00 (ref)	1.00 (ref)	1.00 (ref)	1.00 (ref)
36-45 years	2.49 (1.41 to 4.38)**	1.92 (1.08 to 3.42)*	1.76 (0.98 to 3.16)	1.83 (1.01 to 3.3)*
46-55 years	5.26 (3.02 to 9.15)**	2.63 (1.46 to 4.76)**	2.4 (1.3 to 4.42)**	2.2 (1.21 to 4.01)*
56-65 years	7.52 (4.14 to 13.67)**	2.82 (1.45 to 5.46)**	3.11 (1.58 to 6.13)**	2.26 (1.13 to 4.54)*
≥66 years	11.89 (6.26 to 22.60)**	3.76 (1.79 to 7.9)***	3.01 (1.31 to 6.87)**	2.41 (1.12 to 5.19)*
Sex				
Female	1.00 (ref)	1.00 (ref)	1.00 (ref)	1.00 (ref)
Male	4.92 (3.05 to 7.95)**	3.17 (1.92 to 5.23)***	3.4 (1.95 to 5.95)***	3.79 (2.25 to 6.36)**
Townsend Deprivation Quintile				
First (least deprived)	2.06 (1.09 to 3.9)*	1.97 (1.01 to 3.85)*	1.98 (0.96 to 4.09)	1.84 (0.92 to 3.67)
Second	1.15 (0.59 to 2.24)	1.25 (0.63 to 2.47)	1.17 (0.55 to 2.48)	1.16 (0.55 to 2.43)
Third	1.00 (ref)	1.00 (ref)	1.00 (ref)	1.00 (ref)
Fourth	1.11 (0.64 to 1.93)	1.24 (0.71 to 2.18)	1.13 (0.61 to 2.07)	1.23 (0.68 to 2.24)
Fifth (most deprived)	1.34 (0.82 to 2.19)	1.6 (0.96 to 2.68)	1.61 (0.93 to 2.77)	1.63 (0.95 to 2.8)
Ethnicity				
White	1.00 (ref)	1.00 (ref)	1.00 (ref)	1.00 (ref)
Black African	0.74 (0.48 to 1.14)	1.28 (0.78 to 2.08)	1.22 (0.72 to 2.04)	1.09 (0.53 to 2.22)
Bangladeshi	1.29 (0.67 to 2.47)	1.34 (0.66 to 2.72)	1.3 (0.63 to 2.7)	1.2 (0.43 to 3.34)
Caribbean	2.23 (1.01 to 4.89)*	3.15 (1.3 to 7.67)*	3.12 (1.07 to 9.04)*	0.59 (0.32 to 1.09)
Chinese	0.75 (0.44 to 1.28)	1.02 (0.58 to 1.8)	1 (0.56 to 1.81)	1.29 (0.53 to 3.12)
Indian	1.67 (0.82 to 3.38)	2.01 (0.93 to 4.37)	2.21 (0.97 to 5.06)	0.76 (0.41 to 1.41)
Other	0.73 (0.42 to 1.28)	1.21 (0.68 to 2.13)	0.88 (0.46 to 1.71)	0.61 (0.27 to 1.39)
Other Asian	0.59 (0.25 to 1.37)	1.29 (0.54 to 3.1)	1.03 (0.39 to 2.7)	0.68 (0.3 to 1.51)
Pakistani	0.47 (0.2 to 1.1)	1.25 (0.6 to 2.64)	1.39 (0.65 to 2.95)	1.09 (0.53 to 2.22)
Cigarette consumption				
Non-smoker	1.00 (ref)	1.00 (ref)	1.00 (ref)	1.00 (ref)
Ex-smoker	1.1 (0.68 to 1.78)	0.97 (0.61 to 1.55)	1.05 (0.64 to 1.73)	0.63 (0.37 to 1.07)
Light smoker (1-9 cigarettes/day)	1.73 (1.19 to 2.53)**	1.32 (0.87 to 1.98)	1.28 (0.82 to 1.99)	1.18 (0.76 to 1.82)
Moderate to heavy smoker (≥10 cigarettes per day)	1.34 (0.67 to 2.65)	1.65 (0.85 to 3.18)	1.62 (0.78 to 3.38)	1.82 (0.95 to 3.49)
Alcohol consumption				
Non-drinker	1.00 (ref)	1.00 (ref)	1.00 (ref)	1.00 (ref)
Trivial drinker (<1 unit per day)	1.24 (0.78 to 1.98)	1.23 (0.75 to 2.01)	1.23 (0.73 to 2.06)	1.02 (0.57 to 1.82)
Light drinker (1-2 units per day)	0.81 (0.36 to 1.84)	0.75 (0.3 to 1.87)	0.7 (0.25 to 1.94)	0.54 (0.2 to 1.46)
Moderate to heavy drinker (≥3 units per day)	1.26 (0.52 to 3.08)	1.6 (0.75 to 3.41)	1.8 (0.75 to 4.31)	0.63 (0.23 to 1.69)
BMI				
Underweight (<18.5 kg/m^2^)	1.3 (0.56 to 3)	1.34 (0.52 to 3.45)	1.91 (0.73 to 4.95)	0.65 (0.19 to 2.21)
Normal weight (18.5-24.9 kg/m^2^)	1.00 (ref)	1.00 (ref)	1.00 (ref)	1.00 (ref)
Overweight (25.0-29.9 kg/m^2^)	1.07 (0.75 to 1.52)	1.19 (0.83 to 1.71)	1.16 (0.78 to 1.73)	0.86 (0.59 to 1.25)
Obese (≥30 kg/m^2^)	1.06 (0.68 to 1.63)	1.09 (0.69 to 1.74)	1.15 (0.7 to 1.89)	0.79 (0.49 to 1.28)
Antiviral initiation				
No initiation	1.00 (ref)	1.00 (ref)	1.00 (ref)	1.00 (ref)
Initiation during study	3.64 (1.91 to 3.66)**	1.10 (0.78 to 1.57)	1.11 (0.76 to 1.62)	1.5 (0.62 to 2.01)
Type 2 DM	2.57 (1.79 to 3.7)**	1.54 (0.72 to 3.29)	1.04 (0.43 to 2.52)	1.46 (0.63 to 3.37)
Congestive heart failure	2.1 (0.78 to 5.68)	--	--	--
Hypertension	2.32 (1.68 to 3.22)**	--	--	--
Chronic kidney disease	3.83 (2.5 to 5.88)**	1.25 (0.78 to 2.02)	1.26 (0.74 to 2.14)	1.01 (0.61 to 1.68)
Alcohol-related liver disease	5.82 (3.06 to 11.05)**	0.93 (0.44 to 1.95)	1.19 (0.56 to 2.54)	1 (0.45 to 2.22)
Ascites	13.61 (8.42 to 21.99)**	2.28 (1.28 to 4.06)**	2.62 (1.37 to 5)**	1.68 (0.93 to 3.07)
Cerebrovascular disease	2.31 (1.4 to 3.83)**	--	--	
Cirrhosis	12.65 (9.22 to 17.36)**	6.55 (4.57 to 9.38)***	7.47 (5.06 to 11.03)***	5.11 (3.5 to 7.47)**
End-stage liver disease	7.66 (4.34 to 13.52)**	1.32 (0.67 to 2.6)	1.01 (0.46 to 2.25)	1.75 (0.88 to 3.45)
Ischaemic heart disease	3.01 (1.82 to 4.99)**	--	--	--
Non-alcoholic fatty liver disease	0.69 (0.32 to 1.48)	0.62 (0.28 to 1.36)	0.47 (0.19 to 1.18)	0.75 (0.33 to 1.68)
Non-HCC neoplasm	2.3 (1.45 to 3.67)**	0.96 (0.58 to 1.59)	--	
Peptic ulcer	5.54 (3.68 to 8.34)**	2.26 (1.45 to 3.51)***	2.41 (1.5 to 3.86)***	1.96 (1.22 to 3.15)
Any cardiovascular disease§	2.81 (2.05 to 3.84)**	1.4 (0.97 to 2.04)	1.39 (0.93 to 2.07)	1.35 (0.88 to 2.59)
Antidiabetic drug use	2.53 (1.75 to 3.65)**	1.94 (0.8 to 4.72)	2.2 (0.83 to 5.81)	1.09 (0.4 to 2.94)
Antihypertensive use	2.2 (1.39 to 3.48)**	0.48 (0.22 to 1.06)	0.5 (0.21 to 1.23)	0.92 (0.4 to 2.15)
NSAID use	1.96 (1.24 to 3.1)**	0.82 (0.41 to 1.63)	0.75 (0.35 to 1.61)	1.16 (0.54 to 2.49)
Statin use	1.72 (1.06 to 2.78)*	0.53 (0.25 to 1.11)	0.77 (0.33 to 1.78)	0.38 (0.17 to 0.84)
ALT quintile				
First quintile (mean log ALT=, SD=)	--	--	--	0.95 (0.46 to 1.99)
Second quintile (mean log ALT=, SD=)	--	--	--	1.91 (1.03 to 3.56)*
Third quintile (mean log ALT=, SD=)	--	--	--	1.00 (ref)
Fourth quintile (mean log ALT=, SD=)	--	--	--	1.93 (1.08 to 3.46)*
Fifth quintile (mean log ALT=, SD=)	--	--	--	2.34 (1.35 to 4.06)**
AST quintile				
First quintile (mean log AST=, SD=)	--	--	--	0.6 (0.33 to 1.07)
Second quintile (mean log AST=, SD=)	--	--	--	0.99 (0.56 to 1.73)
Third quintile (mean log AST=, SD=)	--	--	--	1.00 (ref)
Fourth quintile (mean log AST=, SD=)	--	--	--	0.84 (0.5 to 1.4)
Fifth quintile (mean log AST=, SD=)	--	--	--	0.97 (0.6 to 1.57)
PL quintile, m (%)				
First quintile (mean log PL=, SD=)	--	--	--	1.25 (0.75 to 2.08)
Second quintile (mean log PL=, SD=)	--	--	--	0.67 (0.36 to 1.23)
Third quintile (mean log PL=, SD=)	--	--	--	1.00 (ref)
Fourth quintile (mean log PL=, SD=)	--	--	--	0.79 (0.41 to 1.52)
Fifth quintile (mean log PL=, SD=)	--	--	--	1.01 (0.56 to 1.84)

HR, hazards ratio; CHB, chronic hepatitis B virus infection; ICD, International classification of disease; SNOMED, Systemised Nomenclature of Medicine; SD, standard deviation; BMI, body mass index; DM, diabetes mellitus; HCC, hepatocellular carcinoma; NSAID, non-steroidal anti-x drug.

* *P* < 0.05

** *P* < 0.01

† Patients with history of any non-hepatocellular carcinoma neoplasm excluded.

‡ AST, ALT and Plt added to model.

§ Includes congestive heart failutre, hypertension, cerebrovascular disease and ischaemic heart disease.

## List of abbreviations

95CI: 95% confidence interval
aHR: Adjusted Hazards Ration
ALT: Alanine aminotransferase
AST: Aspartate transaminase
BMI: Body mass index
CHB: Chronic hepatitis B virus infection
EHR: Electronic health record
HBsAg: Hepatitis B surface antigen
HBV: Hepatitis B virus
HCC: Hepatocellular carcinoma
HES: Hospital episode statistics
HIC: Health Informatics Collaborative
ICD: International Classification of Disease
IQR: Interquartile range
MICE: Multiple imputation by chained equations
NA: Nucleoside analogue
NCRAS: National Cancer Registration Analysis Service
NSAID: Non-steroidal anti-inflammatory drug
ONS: Office for National Statistics
Plt: Platelet
PPI: Proton pump inhibitor
SD: Standard deviation
SNOMED: Systemised Nomenclature of Medicine
T2DM: Type 2 diabetes mellitus
UK: United Kingdom
VL: Viral load
WHO: World Health Organisation
